# Genome-Wide Pathway Analysis Reveals Different Signaling Pathways between Secreted Lactoferrin and Intracellular Delta-Lactoferrin

**DOI:** 10.1371/journal.pone.0055338

**Published:** 2013-01-30

**Authors:** Byungtak Kim, Seongeun Kang, Sun Jung Kim

**Affiliations:** Department of Life Science, Dongguk University-Seoul, Seoul, Korea; University of Jaén, Spain

## Abstract

Human lactoferrin (LF) is a multifunctional protein involved in immunomodulation, cellular growth, and differentiation. In addition to its secreted form (sLF), an alternative form (ΔLF) lacking the signal sequence has been found to be downregulated in cancer. Although the signaling pathways mediated by LF have been studied in a few cell models, there have been no relevant systemic approaches. Therefore, this study was carried out to identify and compare signaling networks provoked by the two LF isoforms. For this, the two forms were overexpressed in HEK293 cells using the Flp-In T-Rex system, after which genome-wide expression analysis of 18,367 genes was conducted. Pathway analysis of the genes showing altered expression identified pathways which are responsible for cell survival and apoptosis. In addition, the pathways mediated by the two LF forms were within distantly related networks. GPCR, PI3K complex, and POU5F1, which are involved in receptor-mediated pathways, were centered in the sLF network, whereas RIF1, NOS3, and RNPS1, which are involved in intracellular signaling, were centered in the ΔLF network. These results suggest that structural differences between the LF isoforms, mainly glycosylation, determine the fate of LF signaling. Furthermore, these findings provide information relating to the role of ΔLF which is downregulated during carcinogenesis.

## Introduction

Lactoferrin is a pleiotrophic, iron-binding glycoprotein which is present in high concentrations in the colostrum [1]. It is also found in most body fluids, including tears, saliva, vaginal fluid, and semen [2]. The principle function of LF is modulation of immune and inflammatory responses by acting on immune cells, and it is secreted from the specific granules of neutrophils [3], [4]. The protein is also involved in the regulation of cell growth and cellular differentiation [1].

One form of LF is secreted in body fluid (sLF), whereas an alternative form (ΔLF), regulated by a different promoter, is present in normal tissues and has been shown to be downregulated in breast cancer [5], [6]. The ΔLF exon 1 is present in the first intron of the LF gene. Apart from the 5′-end, the ΔLF messenger is identical to sLF mRNA. ΔLF is a protein devoid of the 45 first amino acid residues, including the leader sequence, implying that it is a 73 kDa cytoplasmic isoform [5], [7]. However, both ΔLF and sLF have been observed in the nucleus, and a short bipartite nuclear localization signal at the C-terminus has been identified in LFs from different species [8], [9]. The expression level of ΔLF has a high prognostic value for human breast cancer; high concentrations of ΔLF are associated with longer relapse-free and overall survival [6]. These findings suggest that ΔLF may play an important role in the regulation of normal cell growth.

LF isoforms function in host defense and tumor cell growth through the modulation of various transduction pathways. Only sLF is involved in various aspects of host defense mechanisms [1], [10], while both sLF and ΔLF may possess anti-tumoral activities [11]. sLF shows anti-inflammatory activity through the inhibition of pro-inflammatory cytokines such as interferon-γ, tumor necrosis factor-α, interleukin (IL)-1β, IL-2, IL-6, and NK-κB [12], [13]. Relatively low levels of iron-saturated LF, LF(Fe^3+^) are required to stimulate S phase cell cycle entry and initiate Akt activation in MCF-7 cells [14]. The catabolic influence of LF together with a proliferative stimulus enhances MMPs in chondrocytes, necessary for degradation of damaged tissue and stimulating proliferation of chondrocytes during reconstruction [15]. JNK-associated Bcl-2 signaling pathway is mediated by LF during apoptosis of human leukemia Junket T-cells [16] and PC12 neuronal cells [17].

ΔLF provokes antiproliferative effects and cell cycle arrest in S phase by increasing SKP1, a key component of the Skp1/Cullin-1/F-box ubiquitin ligase complex responsible for the ubiquitination of cellular regulators, leading to their proteolysis [9]. DcpS, a key enzyme in mRNA decay, has been identified as a target gene of ΔLF in a proteomic study [7].

Most previous studies concerning the pathway-related function of the two isoforms of LF have been carried out at the level of individual genes or a small group of genes. In this study, we attempted to identify genome-wide signaling pathways that are provoked by sLF and ΔLF. Our findings showed that the two LF isoforms acted on different signaling pathways: sLF signaled through Jnk/Akt/P38 MAPK pathways, whereas ΔLF signaled through the RIF1, NOS3, and RNPS1 pathways.

## Materials and Methods

### Recombinant Flp-In T-Rex plasmid construction

A 2.0 kb full-length human LF cDNA harboring the signal sequence and the entire coding region was amplified from the LF-containing pWL construct [18] by PCR, followed by sub-cloning into the *Hin*dIII and *Bam*HI sites of pcDNA5/FRT/TO plasmid (Invitrogen, Carlsbad, CA) to construct psLF. A 1.9 kb cDNA missing the signal sequence was also amplified by PCR and was used to construct pΔLF. The primers for PCR amplification are listed in Table S1.

### Cell culture, stable transfection, and induction of LF expression

T-Rex human HEK293 cells were purchased from Invitrogen and were cultured in DMEM medium supplemented with 10% fetal calf serum and penicillin-streptomycin. To establish stable cell lines expressing human LF, 2 μg each of LF-expressing vector and pOG44 (Flp recombinase) was transfected into 1×10^6^ of HEK293 cells using Effectene (Qiagen, Valencia, CA), according to the manufacturer's instructions, and the cells were grown in a 75 cm2 culture flask. As a control, pcDNA5/FRT/TO without LF cDNA was transfected with pOG44. Two days after transfection, hygromycin (200 μg/ml) (GibcoBRL, Carlsbad, CA) was added to select stable transfectants formed by Flp-In recombination. Stable transfectants resistant to hygromycin were selected to monitor LF expression by RT-PCR and Western blot analysis. To induce LF expression from recombinant Flp-In cells, the cells were treated with tetracycline for 24 and 36 h at 1.0 μg/ml prior to harvesting.

### RT-PCR analysis

Total RNA from stably transfected cells was isolated using Trizol (GibcoBRL, Carlsbad, CA) on a Ribolyser cell disruptor (Qbio, Carlsbad, CA), following the supplier`s protocol. First-strand cDNA was synthesized from total RNA using a Reverse Transcription System kit (Promega, Madison, WI) according to the instructions of the manufacturer. Expression was determined by end-point and real-time RT-PCR. End point RT-PCR was conducted by subjecting 1 μl of the samples to 25 cycles of 94°C for 45 sec, 62°C for 1 min, and 72°C for 40 sec. Then, 1 μl of cDNA was used for quantitative real-time PCR, and duplicate reactions were conducted for each sample using a Kapa SYBR Fast qPCR Kit (Kapa Biosystems, Woburn, MA) with gene-specific primers on an ABI 7300 thermal cycler (Applied Biosystems, Foster City, CA). The primers used are listed in Table S1. The RNA quantity was normalized against the GAPDH content, and gene expression was quantified according to the 2^−ΔCt^ method.

### Microarray analysis

Approximately 1×10^7^ proliferating HEK293 cells stably transfected with recombinant psLF and pΔLF plasmids, or the vector alone were harvested, and total RNA was extracted using an RNeasy mini kit (Qiagen). The RNA was then forwarded to microarray analysis in duplicate arrays of a chip (Digital-Genomics, Korea) containing 18,367 human cDNAs and ESTs. Clones that were differentially regulated in both experiments with a significant ratio of Cy3 to Cy5 (defined as greater than the value of 2) were selected and further analyzed. All array data have been uploaded to the Gene Expression Omnibus (GEO) database, and can be accessed via its website (http://www.ncbi.nlm.nih.gov/geo/) under the accession number GSE31878.

### Immuno blot analysis and ELISA

For Western blot analysis, total proteins from stably transfected cells in 75 cm^2^ culture flask were extracted using 400 μl of the Pro-PREP solution (Intron, Korea). 50 µg of protein was loaded onto an 8% denaturing polyacrylamide gel. After electrophoresis, proteins were transferred to a nitrocellulose membrane. Polyclonal rabbit anti-human LF antiserum (Sigma, St. Louis, MO) and HRP (Horseradish peroxidase)-conjugated goat anti-rabbit IgG (Sigma) were diluted 1∶5,000 and 1∶1,000 with 1% bovine serum albumin, respectively and were used to detect LF. Bound antibody was detected by addition of West-Zol (Intron). For ELISA analysis, 50 μl of culture media was mixed with 250 μl of PBS and the LF levels were determined using an hLF ELISA kit (Calbiochem, Billerica, MA).

### Pathway analysis

To identify the pathways displaying altered expression patterns with potential roles in LF signaling, functional categorization and pathway construction were performed using the Ingenuity Pathway Analysis (IPA) software tool produced by Ingenuity Systems. IPA utilizes an extensive database of functional interactions, which are drawn from peer-reviewed publications and are manually maintained [19]. Scores for individual networks were obtained by comparing the likelihood of obtaining the same or a higher number of transcripts in a random gene set as that actually present in the input file (i.e., the set of genes differentially expressed in LF-expressing 293 cells) using a Fischer's exact test, based on hypergeometric distribution. The functional network with the highest confidence was designated as the top network.

### Statistical analysis

From the microarray data, observations with adjusted p-values ≥0.05 were removed and were precluded from further analysis. Adjustments were made to control for false discoveries. A multiple comparisons correction was applied to each observation using the Benjamini-Hochberg method, as previously described [20], to obtain a false discovery rate–adjusted *P* value. Following adjustment, the remaining genes were defined as differentially expressed if they displayed at least a 2-fold difference in expression between the control and sLF/ΔLF groups, in order to further reduce the number of false positive observations and to enrich the biologically relevant expression changes. For the analysis of RT-PCR, *t*-Test was used with *P*-values below 0.05 considered to be statistically significant. All statistical analyses were performed using SPSS software, version 17.0 (SPSS Inc., Chicago, IL).

## Results

### Establishment of Flp-In 293 cells expressing human LF

**Figure 1:**
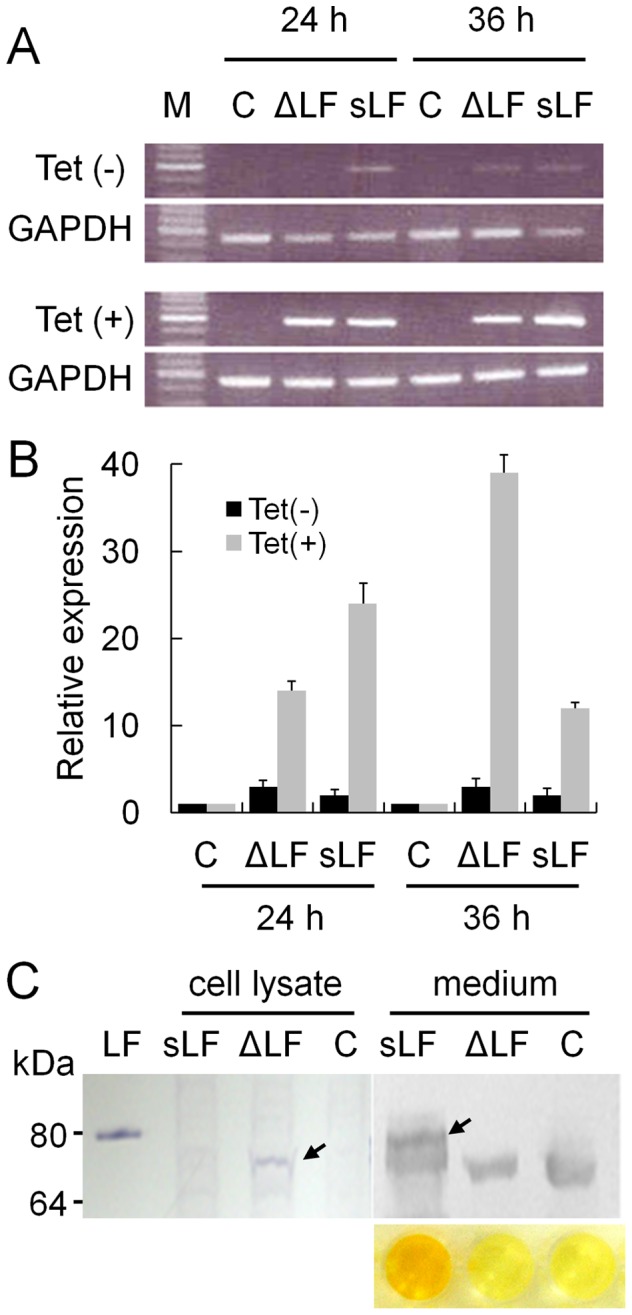
Overexpression of human LF in HEK293 cells using Flp-In T-Rex system. (A) End-point RT-PCR analysis of LF. RT-PCR was conducted for RNAs from HEK293 cells transfected with T-Rex LF expression vectors with signal sequence (sLF) or without signal sequence and 26 N-terminal amino acid residues (ΔLF), non-treated with (Tet(−), top panel) or treated with tetracycline (Tet(+), bottom panel) for 24 or 36 h. C, vector alone. M, size marker. (B) Real-time RT-PCR analysis of LF. Each reaction per clone was carried out in duplicate, and the average of 15 clones is presented along with the standard error. (C) Immunoblot analysis of LF. LF protein expressed from recombinant vector was immunobloted using anti-LF antibody from cell lysate and culture media. LF, commercially available sLF (80 kDa). C, vector alone. LFs expressed from vectors with and without the signal sequence and 26 N-terminal amino acid residues are marked by arrows. Signals below the LF bands appearing in all samples in the medium are non-specifically-reacted serum proteins. The result of ELISA for the LF in medium is shown at the bottom.

To explore and compare the effects of the two forms of LF, sLF and ΔLF, on gene expression and functional pathways, the proteins were overexpressed in HEK293 cells using the Flp-In recombination system, which express LF receptor (http://www.abcam.com/ITLN1-antibody-17-ab101101.html). As the two forms of LF, secreted form (sLF) and intracellular form (ΔLF), are produced by different promoter and show differential expression in normal and cancer cells, we aimed to compare the signaling pathways initiated by the two proteins. For this, two LF expression vectors encoding sLF and ΔLF were constructed in pcDNA5/FRT/TO and were stably transfected into HEK293 cells. The vector alone was also transfected as a control. Integration of LF cDNA into the chromosomal DNA of H293 cells was confirmed through amplification of a LF-specific product by PCR (data not shown). RT-PCR was carried out for each of 15 clones of sLF and ΔLF, and Western blot analysis was carried out for collections of more than 100 clones. When the cells harboring sLF or ΔLF were treated with tetracycline for 24 and 36 h, they both expressed LF RNA with a 4∼15-fold increase compared to those of non-treated cells. In contrast, cells harboring the control vector did not express LF RNA (Fig. 1A and B). PCR from RNA itself produced a negligible amount of amplified product confirming the lack of genomic DNA contamination. Western blot analysis identified that the ΔLF form was present only in the cell lysate with a smaller size of approximately 73 kDa than that of sLF due to the lack of glycosylation and the 26 N-terminal amino acid residues of sLF (Fig. 1C). Expression of sLF was observed only in the culture media, showing the same size as LF purified from milk. A band below sLF that also appeared in ΔLF and vector alone was the result of a non-specific reaction between the antibody and a serum protein. ELISA analysis of the culture media identified specific expression of the protein only in LF cells (Fig. 1C).

### Genome-wide expression analysis shows that secreted and intracellular LF acts on different signaling pathways

**Figure 2:**
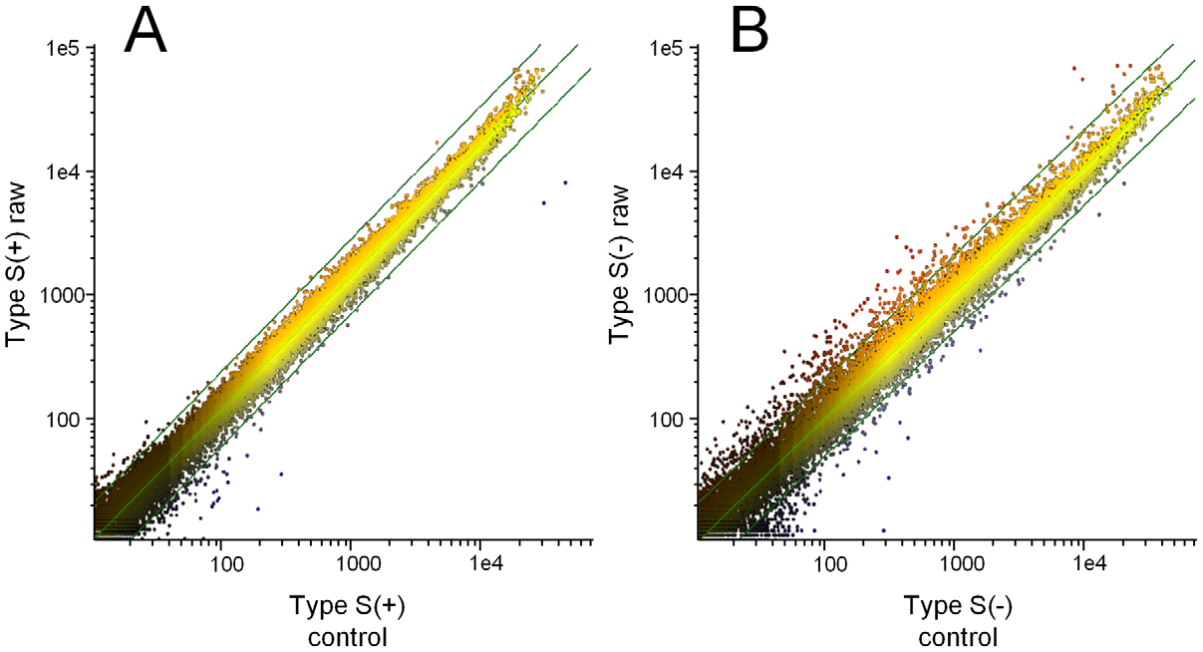
Genome-wide expression analysis in LF-overexpressing HEK293 cells. Expression histogram of control (X-axis) vs. sLF (Y-axis) (A) and control vs. ΔLF (B). Expression levels of 18,367 genes were measured by Phalanx Human 32K microarray and are presented on a log scale. Best fit and two-fold difference lines were added. Altered expression was observed more often in ΔLF cells than in sLF cells, indicating that ΔLF generally regulated more genes.

Genome-wide expression analysis was conducted using the RNAs isolated from a collection of more than 100 LF-overexpressing cell clones. After removing observations with adjusted p-values ≥0.05, the remaining genes were further selected if they displayed at least a 2-fold difference in expression between the control and sLF/ΔLF groups. 74 and 125 genes in sLF cells and 327 and 256 genes in ΔLF cells showed significant up- and downregulation, respectively. The ΔLF cells showed a higher number of genes with altered expression (Fig. 2). As expected, genes previously known to be upregulated by LF [7], [21], including Skp1 (2.7-fold) in sLF and Skp1 (2.6-fold) and TCPB (2.8-fold) in ΔLF, were also upregulated in our sample (Table S2 and S3). The genes with the most altered expression levels were the *FSCN1* gene (9.9-fold decrease) in sLF and the *HSPA8* gene (4.9-fold increase) in ΔLF. FSCN1 is an actin-binding protein related to the development of various tumors [22]. HSPA8 is a member of the heat shock protein family and is known as a cell surface marker of human embryonic stem cells [23].

**Figure 3:**
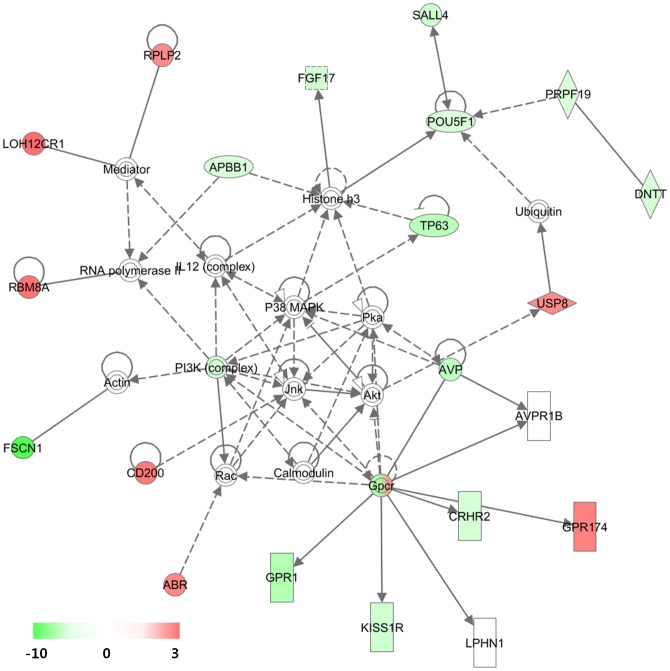
Highest confidence network of genes regulated by sLF. Highest confidence network of genes displaying altered expression levels in response to secreted sLF in HEK293 cells. According to IPA, the network is relevant to ‘Genetic Disorder, Hematological Disease, Metabolic Disease’. Genes that were upregulated are presented in red, whereas those that were downregulated are presented in green, where the intensity of the color reflects the magnitude of the expression change, as indicated in the scale bar. Each interaction is supported by at least one literature reference, with solid lines representing direct interactions and dashed lines representing indirect interactions.

Regarding the top IPA networks constructed by the sLF-regulated 199 genes, the highest functional network resulting from differential gene expression was designated as “Genetic Disorder, Hematological Disease, and Metabolic Disease” (Fig. 3 and Fig. S1). Interestingly, GPCR, PI3K complex, and POU5F1 were linked with many other genes with altered expressions (Fig. 3). In the network, one GPCR (GPR174) was upregulated while two (GPR54 and GPR1) were downregulated. Iron-saturated LF is able to stimulate cell cycle progression through the PI3K/Akt pathway [14], and our results indicate that LF regulated the expression of genes involved in PI3K/Akt signaling. Downregulation of POU5F1 (OCT4) by LF was first identified in this study, and this result might be correlated with its role in maintaining cells in a differentiated state [24]. It is notable that SALL4, a recently identified stem cell factor [25], was also downregulated in the network. In addition, another target gene of OCT4, ZFHX3 that has been known to play a role in cellular differentiation [26], was upregulated (1.4-fold increase).

Jnk, Akt, and P38 MAPK were also positioned at the hub in the network, being linked with GPCR and PI3K complex. However, these elements were not altered in expression, as determined by microarray analysis. This result coincides with previous studies in which their gene activities were affected by phosphorylation and not LF expression [14]. These three proteins are key signaling molecules in proliferation, apoptosis, and differentiation [27]. A previous study showed that LF-induced gene activation of AP-1 is mediated via the JNK and p38 MAPK pathways [28].

**Figure 4:**
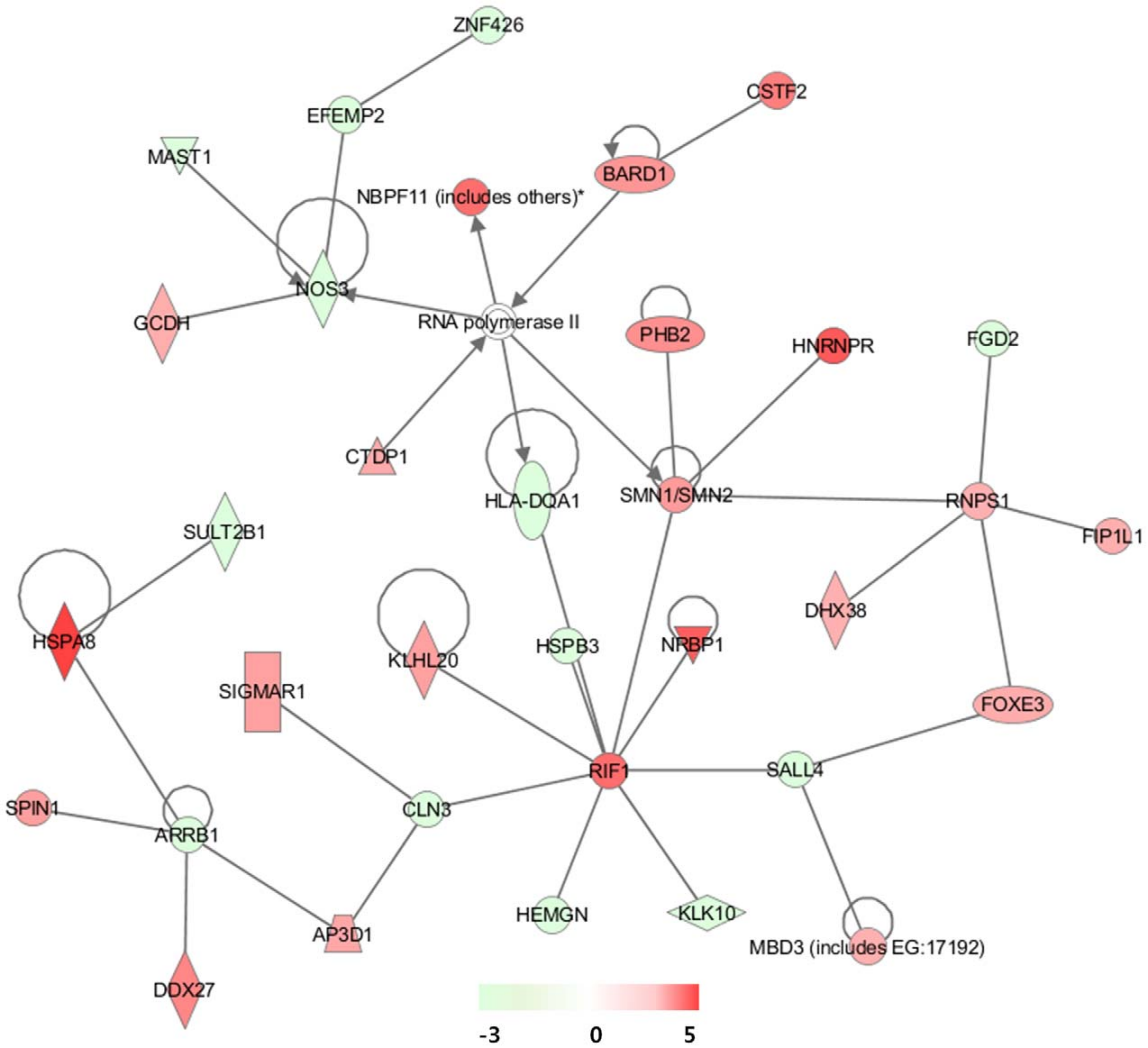
Highest confidence network of genes regulated by ΔLF. Highest confidence network of genes displaying altered expression levels in response to intracellular ΔLF in HEK293 cells. According to IPA, the network is relevant to ‘RNA Post-Transcriptional Modification, Embryonic Development, Tissue Development’.

The IPA analysis of the ΔLF-regulated 583 genes showed far-related networks with a few elements in common with the sLF networks (Fig. 4 and Fig. S2). The highest functional network resulting from differential gene expression was designated as “RNA Post-Transcriptional Modification, Embryonic Development, Tissue Development”. Namely, RIF1, NOS3, and RNPS1 were notably linked with other genes (Fig. 4). RIF1 (3.78-fold increase) has been implicated in a wide variety of cellular processes in mammals, including pluripotency of stem cells, response to double-strand breaks, and breast cancer development [29]. sLF has been implicated in the activation of the endogenous opioidergic system via nitric oxide synthase activation [30]. The downregulation of NOS3 (2.5-fold decrease) implies that ΔLF regulated the system in part by altering the level of NO. RNPS1 is not only a regulator of alternative splicing but may also play a more fundamental role as a general activator of pre-mRNA splicing [31], [32]. In the context of RNA splicing, SMN, HNRNPR, and DHX38 from the IPA category are pre-mRNA splicing-promoting proteins. SMN is a part of the multiprotein spliceosome functioning in pre-mRNA splicing and HNRNPR acts a SMN interaction partner [33]. DHX38 is DEAH-box ATPase required for the second step of splicing [34].

**Figure 5:**
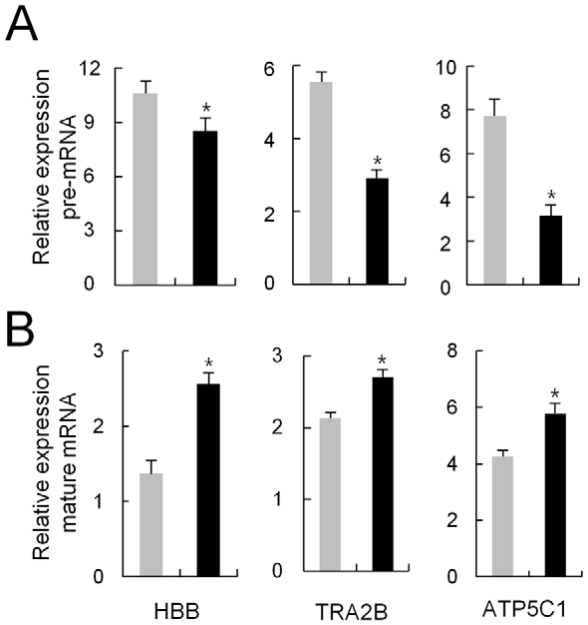
Pre-mRNA levels in ΔLF-overexpressing cells. Real-time RT-PCR was carried out to measure the pre-mRNA (A) and mature-mRNA levels (B) of the three selected genes. Primers spanning an exon and its downstream intron were used for pre-mRNA and primers spanning two exons were used for mature-mRNA. Gray and black bars denote the expression of the indicated genes for non-treated and treated with tetracycline in the ΔLF cells, respectively. Each clone was analyzed in duplicate, and the average relative expression level of 15 clones is presented with the standard error. *Significant (P<0.05; Student's *t*-test).

To further verify that ΔLF was related to “RNA Splicing”, its role in RNA processing was examined by comparing pre-mRNA and mature-mRNA levels of the randomly selected HBB (1.7-fold increase in the microarray), TRA2B (1.6-fold increase), and ATP5C1 (1.9-fold increase) in 15 ΔLF-overexpressing cell clones. In all the three genes, the pre-mRNA levels were decreased, while the mature-mRNA levels were increased in the tetracycline-induced ΔLF-overexpressing cells compared to the non-induced cells (Fig. 5). The DNase-treated RNA itself did not produce any significant amplification curves implying no attribution to genomic DNA for the synthesis of cDNA.

### Expression confirmation of the differentially expressed genes

**Figure 6:**
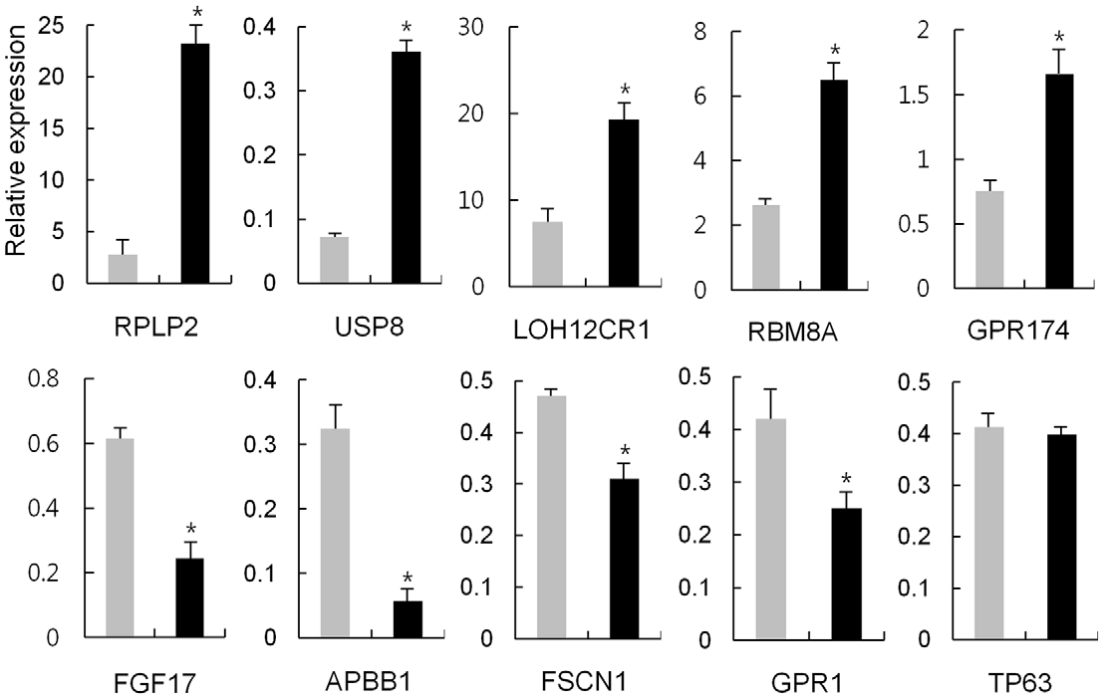
RT-PCR analysis of selected genes induced by sLF. Real-time RT-PCR analysis of ten selected genes displaying altered expression in response to sLF in HEK293 cells. Gray and black bars denote the expression of the indicated genes for non-treated and treated with tetracycline in the sLF cells, respectively. Each sample was analyzed in duplicate, and the average relative expression level of 15 clones is presented. *Significant (P<0.05; Student's *t*-test).

**Figure 7:**
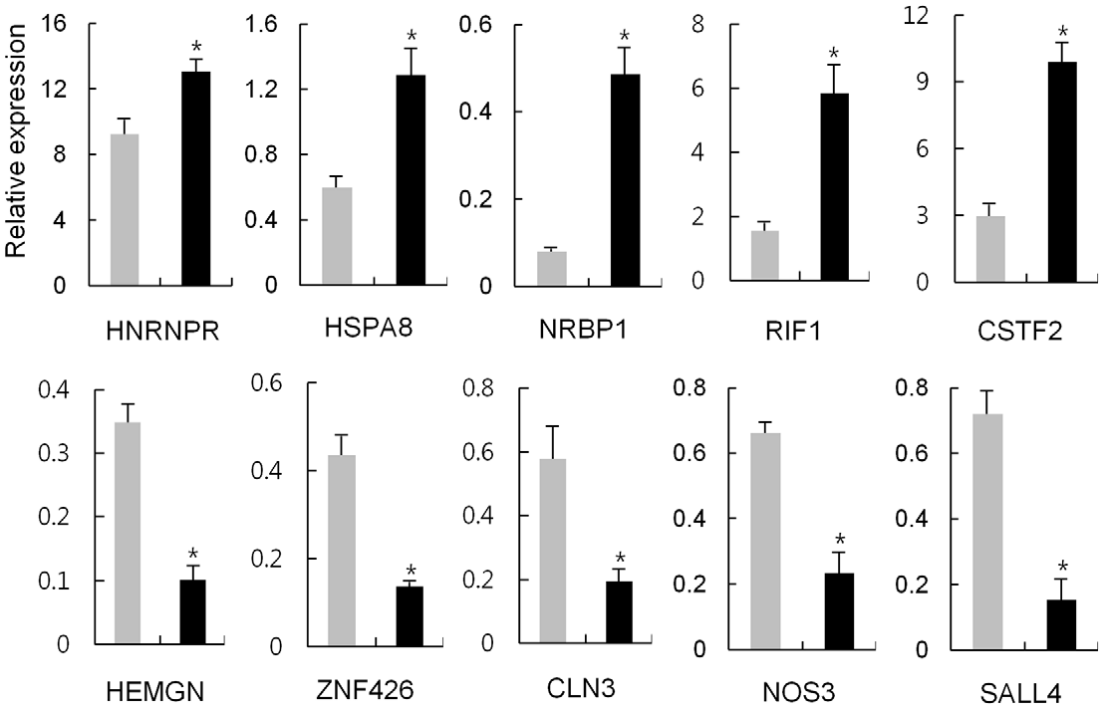
RT-PCR analysis of selected genes induced by ΔLF. Gray and black bars denote the expression of the indicated genes for non-treated and treated with tetracycline in the ΔLF cells, respectively. Each sample was analyzed in duplicate, and the average relative expression level of 15 clones is presented. *Significant (P<0.05; Student's *t*-test).

The altered gene expression in the IPA pathways involved in LF signaling was verified by using real-time RT-PCR to examine the expression levels of selected genes in LF-expressing HEK293 cells. Twenty genes in total, ten from each sLF and ΔLF cells, were selected. In each group five genes were from upregulated, whereas the other five were from downregulated, as determined by microarray. As shown in Fig. 6 and 7, all 20 genes, except for TP63, showed consistent results between RT-PCR and microarray, although the fold differences were not identical. This fact implies that expression levels of genes monitored by the microarray-based expression system generally coincided with that monitored by quantitative RT-PCR, validating the suitability of microarray for determining the effect of LF on gene expression.

## Discussion

This study was conducted to identify the molecular pathways signaled by LF at a genome-wide level and to compare the pathways mediated by the two forms of LF: sLF and ΔLF. The majority of studies on the molecular response of cells to LF have been conducted by treating cells with LF protein in culture media. Through these approaches, sLF has been shown to activate immune cells and to modulate several cytokines, such as tumor necrosis factor (TNF), interleukin (IL)-8, and IL-12 [35]. In this signaling process, sLF directly activates NF-κB, which acts as the master regulator of immune and inflammatory responses. sLF has a highly glycosylated moiety and has been implicated in NF-κB activation [13]. Other master regulators in the pathways controlled by LF are Jnk, Akt, and PI3K complex in MCF7 cells [14]. It is notable that our pathway analysis of sLF in HEK293 cells included the Jnk/Akt/PI3K pathway rather than the NF-κB pathway. Although Jnk, Akt, and P38 MAPK were included in the IPA pathway, their expression was not accompanied by any significant change in expression observed through microarray, suggesting that an alternative regulation might be modulated by LF via phosphorylation without affecting their expression.

In the IPA pathway of sLF, it was revealed that a few GPCRs interacted with Akt. GPCRs cannot be considered as LF receptors (LFRs) since all known LFRs thus far are either LDL receptor-related proteins (LRPs) or asialoglycoprotein (ASGP) receptors, neither one of which is structurally related to GPCR [36]. Therefore, the expression of GPCRs was altered after sLF bound to its membrane receptor, and the expressed GPCRs acted as receptors for other signaling molecules. Upregulated GPCR, GPR174, is an orphan receptor that is overexpressed in metastatic melanoma and contributes to tumor cell survival [37]. On the other hand, downregulated GPCR, GPR54 (also called KISS1R), has been shown to be a metastasis suppressor in numerous cancers in humans [38]. However, recent studies have demonstrated that an increase in GPR54 expression in human breast tumors correlates well with higher tumor grade and metastatic potential [39].

ΔLF was first identified as an alternatively spliced form with sequences replacing the N-terminal peptide sequence of sLF mRNA [5]. ΔLF is a transcriptional factor and is deregulated in cancer cells [9]. Thus, ΔLF is a cytoplasmic protein able to enter the nucleus, although sLF is also found in nucleus [8], [9]. Only sLF is involved in various aspects of host defense mechanism, whereas both sLF and ΔLF may possess anti-tumoral activities [11]. Overexpression of Skp1 provokes cell cycle arrest along with antiproliferative effects, and it has been shown that the Skp1 gene promoter is a target of ΔLF [9]. Skp1 belongs to the Skp1/Cullin-1/F-box ubiquitin ligase complex, which is responsible for the ubiquitination and proteasomal degradation of numerous cellular regulators. A proteomic approach following overexpression of ΔLF in HEK293 cells was able to identify proteins involved in processes such as mRNA maturation and stability, cell viability, proteasomal degradation, and protein and mRNA quality control [7]. Among these proteins, only DcpS and TCPB were also upregulated at the mRNA level. Our microarray array data showed that essential genes responsible for anti-proliferation or anti-apoptosis were dysregulated. For example, RIF1 (3.8-fold increase), which was a sub-hub in the IPA pathway, is an anti-apoptotic factor required in DNA repair [40]. Another sub-hub gene, RNPS1 (2.0-fold increase), controls nonsense-mediated mRNA decay (NMD), which ensures rapid degradation of mRNAs containing premature translation termination codons (PTCs) in eukaryotes [41]. Meanwhile, NOS3, which is involved in cellular proliferation during angiogenesis, showed a 0.4-fold increase in expression, implying an anti-proliferative role for ΔLF. Altogether, both sLF and ΔLF may provoke cell proliferation or apoptosis, depending on the target gene pathway, even in the same cell type.

So far, only a few genes including *SKP1, BAX, DCPS*, and *SELH* promoters have been identified as the target genes of ΔLF [42]. However, any consensus sequence of ΔLF-binding was not found at the promoter region of the genes, *HBB, TRA2B* and *ATP5C1* of which pre-mRNA and mature mRNA level was examined in this study. Thus, it is speculated that ΔLF might be involved in RNA splicing rather than transcriptional factor among the diverse activities at least for the examined genes.

The lower number of genes altered by sLF compared to those altered by ΔLF could have been due to the mechanism of action of sLF, in which sLF is first secreted into the media and then acts on the cell via its receptor. Therefore, the efficacy of sLF is limited by the number of cell surface receptors. Of the genes in the top pathways, SALL4 was common to both sLF (2.73-fold decrease) and ΔLF (2.10-fold decrease). SALL4, a transcription factor that plays an essential role in the embryonic development and self-renewal of embryonic stem (ES) cells, is upregulated in cancer, including breast [43] and lung cancers [44]. In the future, it will be interesting to elucidate the molecular mechanism of LF, specifically as to whether or not it is involved in carcinogenesis by downregulating SALL4.

In summary, we present the genome-wide expression profiles provoked by two forms of LF, sLF and ΔLF, after their overexpression using the Flp-In T-Rex system. Our findings provide new insights into the molecular pathways responsible for cellular proliferation and apoptosis. Further investigation into the mechanisms leading to the differential expression observed with sLF and ΔLF may provide additional information that could prove useful in estimating the role of LF in carcinogenesis.

## Supporting Information

Figure S1 **Pathways most strongly associated with the significantly altered genes in the sLF-expressing HEK293 cells.** (A) Top functional categories. (B) Canonical pathways. The Ingenuity software assigns a *P* value based on the likelihood of obtaining the observed number of category or pathway-related molecules in a given data set by chance alone.
(TIF)

Figure S2 **Pathways most strongly associated with the significantly altered genes in the ΔLF -expressing HEK293 cells.** (A) Top functional categories. (B) Canonical pathways.
(TIF)

Table S1 **Sequences of primers employed in this study.**
(DOC)

Table S2 **Genes in top network displaying differential expression in the sLF expressing cells.**
(DOC)

Table S3 **Genes in top network displaying differential expression in the ΔLF expressing cells.**
(DOC)
