# Supplementary figures and images for: Genome-Wide Pathway Analysis Reveals Different Signaling Pathways between Secreted Lactoferrin and Intracellular Delta-Lactoferrin
Source: PLoS One. 2013 Jan 30;8(1):e55338. doi: 10.1371/journal.pone.0055338 (PMC3559342; doi:10.1371/journal.pone.0055338)

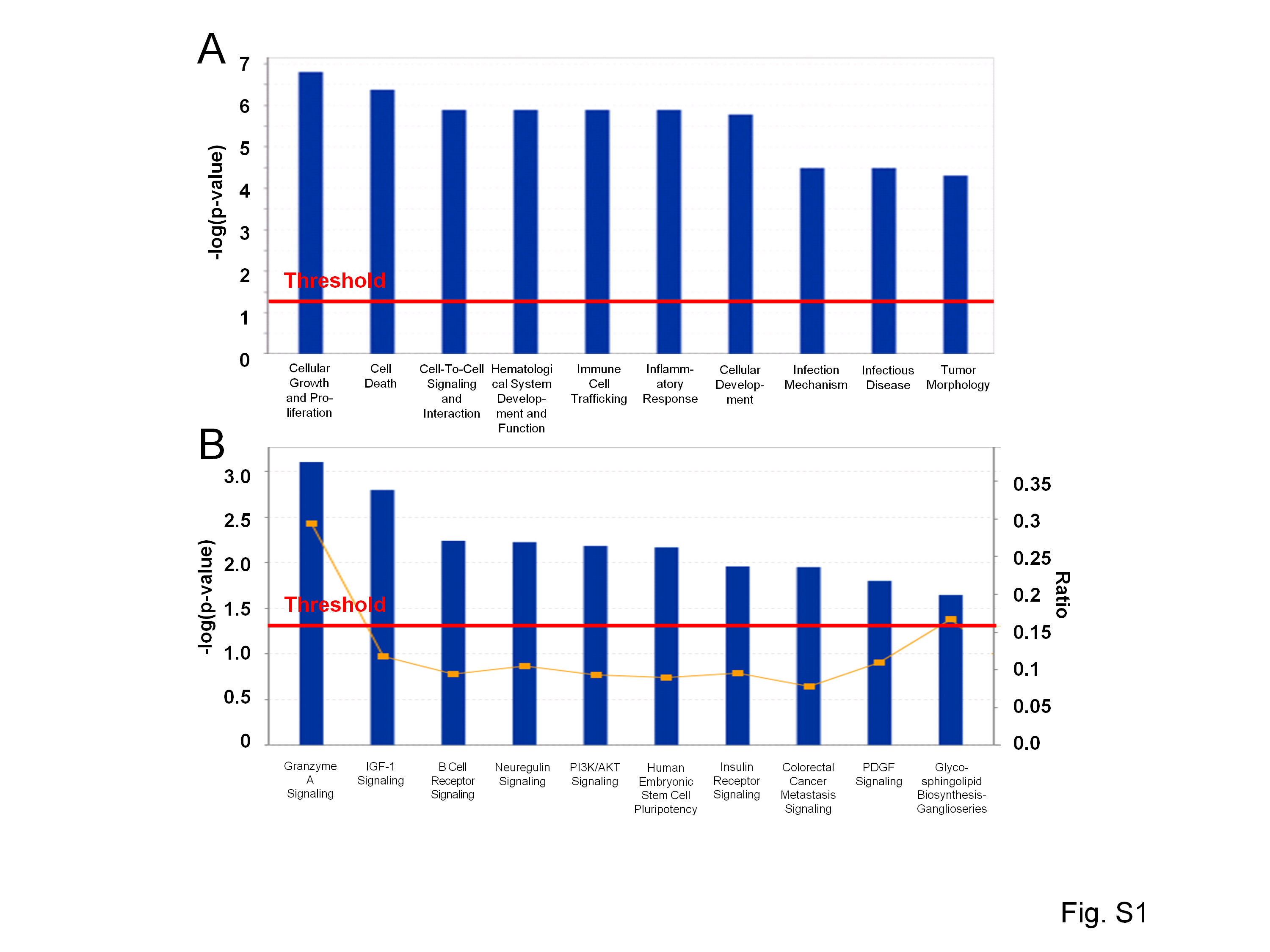

Supplement: Figure S1 — Pathways most strongly associated with the significantly altered genes in the sLF-expressing HEK293 cells. (A) Top functional categories. (B) Canonical pathways. The Ingenuity software assigns a P value based on the likelihood of obtaining the observed number of category or pathway-related molecules in a given data set by chance alone. (TIF) [file pone.0055338.s001.tif]

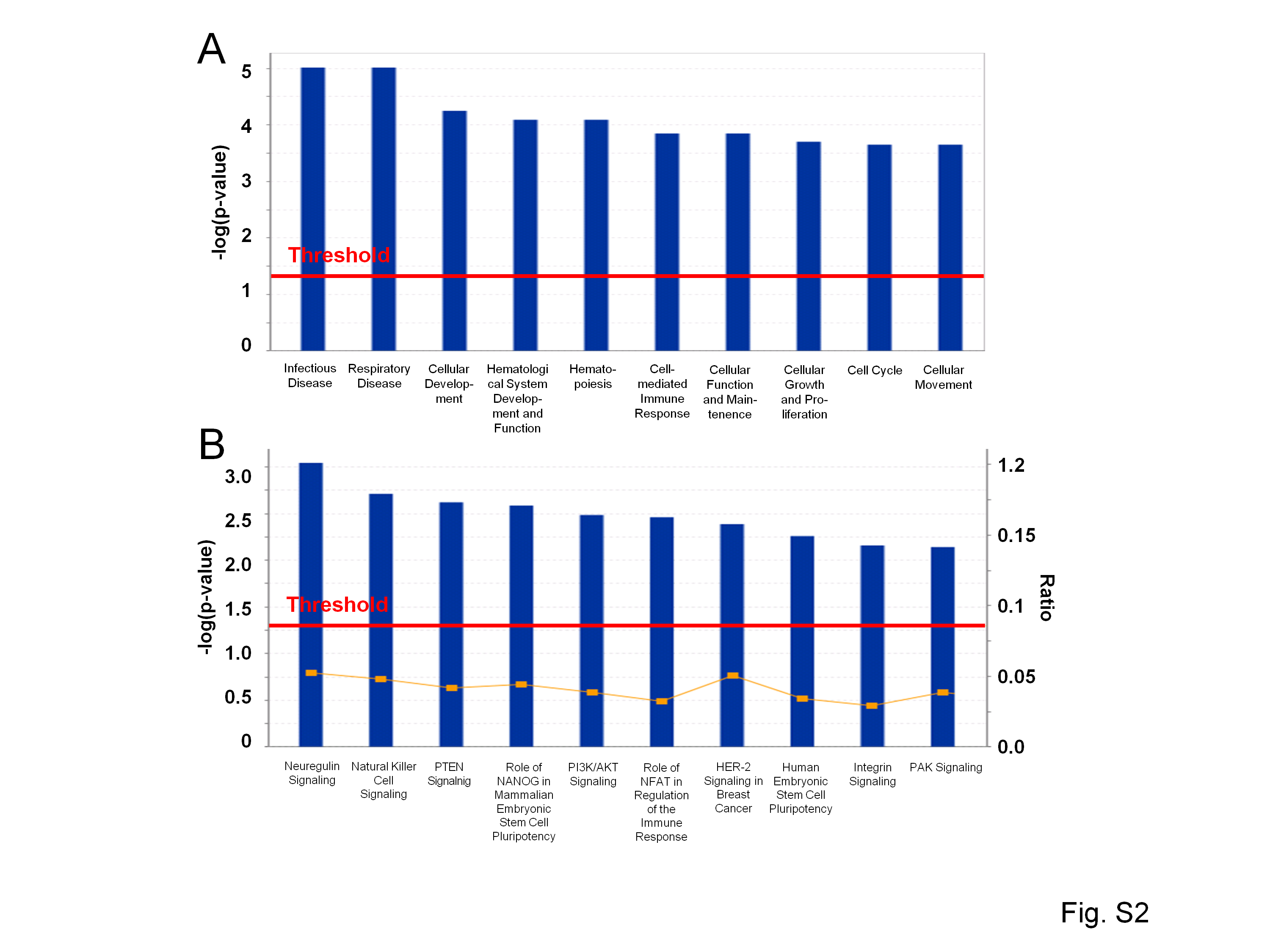

Supplement: Figure S2 — Pathways most strongly associated with the significantly altered genes in the ΔLF -expressing HEK293 cells. (A) Top functional categories. (B) Canonical pathways. (TIF) [file pone.0055338.s002.tif]
