# Supplementary material for: Genome-Wide Pathway Analysis Reveals Different Signaling Pathways between Secreted Lactoferrin and Intracellular Delta-Lactoferrin
Source: PLoS One. 2013 Jan 30;8(1):e55338. doi: 10.1371/journal.pone.0055338 (PMC3559342; doi:10.1371/journal.pone.0055338)
Supplement: Table S1 — Sequences of primers employed in this study. (DOC) [file pone.0055338.s003.doc]

| **Table S1. Sequences of primers employed in this study.** | | | | |
| --- | --- | --- | --- | --- |
| **Genes** | **Forward primer (5’–3’)** | | | **Reverse primer (5’–3’)** |
| **LF expression vecotr** | | |  |  |
| sLF | | GGTTTAGAAGATTTTTTTCGGAATC | | TAAACCCTACACCTTCTATCTCGAT |
| ΔLF | | TTTAGAAGATTTTTTTTGGAATTGG | | TAAACCCTACACCTTCTATCTCAAT |
| **End-point RT-PCR** | |  | |  |
| sLF | | GGTTGGTAGGGAGATAGTATAG | | ATAATTCCCCAACCTAACCTCA |
| ΔLF | | TAGTATAGGAAGGTTTTAGAGT | | CTAACCTCACCTTTCATTCACC |
| GAPDH | | ACCACAGTCCATGCCATCAC | | TCCACCACCCTGTTGCTGTA |
| **Real-time RT-PCR** | |  | |  |
| RPLP2 | | CCCAGGGTATTGGCAAGC | | TCATCTTTCTTCTCCTCTGCTG |
| USP8 | | GAAAGGAGCAATCACAGCAAA | | ACTCCTGGACTGATGGCTTC |
| APBB1 | | GGGGGAAGGAAAGGATCTG | | GCAAAGTCCCTTCCACTGTC |
| FSCN1 | | GCAAGAATGCCAGCTGCTAC | | CTCTGAGTCCCCTGCTGTCT |
| TP63 | | TGAATTCAACGAGGGACAGA | | GTGAATTCAGTGCCAACCTG |
| HNRNPR | | TTTCAGACAGGATTGGTAGCTT | | AGTCACTTTCCTTGAACTGCTG |
| HSPA8 | | AAGCCTACCTTGGGAAGACTG | | CGTAAGCAATAGCAGCAGCA |
| NRBP1 | | AGAGGCGAGAAGAGGTGAATC | | ACAGCACGAACCTTTTCCTC |
| NOS3 | | TTCCGCTACCAGCCAGAC | | AGATCTTCACGGCGTTGG |
| SALL4 | | AGATGGAAGGTCGGAGCAGT | | TGCTAACAAAGGGGTCATCC |
| HBB | | GCAGGCTGCTGGTGGTCTA | | CATAACAGCATCAGGAGTGGA |
| HBB(pre-mRNA) | | TCCTGAGAACTTCAGGGTGAG | | AACGATCCTGAGACTTCCACA |
| TRA2B | | GACCAGCAGTCTAGGCGTTC | | CGAGAGCTGCCATAGGTAGG |
| TRA2B(pre-mRNA) | | GATGCCAAGGAAGTAAGTAAAAGC | | CTTGAGTCTCCACAAGAGGTTG |
| ATP5C1 | | GACCAGTTTCTGGTGGCATT | | TCTTATAGGAGATGACAGACCTGAA |
| ATP5C1(pre-mRNA) | | TTGCAAGTGCTGGTAAGTAGTTTT | | AGATGGTGCCACTGCACTC |
