# Supplementary material for: Genome-Wide Pathway Analysis Reveals Different Signaling Pathways between Secreted Lactoferrin and Intracellular Delta-Lactoferrin
Source: PLoS One. 2013 Jan 30;8(1):e55338. doi: 10.1371/journal.pone.0055338 (PMC3559342; doi:10.1371/journal.pone.0055338)
Supplement: Table S2 — Genes in top network displaying differential expression in the sLF expressing cells. (DOC) [file pone.0055338.s004.doc]

| **Table S2. Genes in top network displaying differential expression in the sLF expressing cells** | | | | |  |  |
| --- | --- | --- | --- | --- | --- | --- |
| **Symbol** | **Accession** | **Description** | **Fold change** | | | |
| *ABR* | NM_001092.3 | Active BCR-related gene | | 2.10 | |  |
| *APBB1* | NM_001164.2 | Amyloid beta (A4) precursor protein-binding, family B, member 1 (Fe65) | | -2.14 | |  |
| *AVP* | AF031475 | Arginine vasopressin (neurophysin II, antidiuretic hormone, diabetes insipidus, neurohypophyseal) | | -3.52 | |  |
| *CD200* | NM_001004197.1 | CD200 antigen | | 2.34 | |  |
| *CRHR2* | NM_001883.2 | Corticotropin releasing hormone receptor 2 | | -2.29 | |  |
| *DNTT* | NM_004088.2 | Deoxynucleotidyltransferase | | -2.23 | |  |
| *FGF17* | NM_003867.1 | Fibroblast growth factor 17 | | -2.52 | |  |
| *FSCN1* | NM_003088.2 | Fascin homolog 1, actin-bundling protein (Strongylocentrotus purpuratus) | | -9.90 | |  |
| *GPR1* | NM_005279.2 | G protein-coupled receptor 1 | | -4.37 | |  |
| *GPR174* | NM_032553.1 | G protein-coupled receptor 174 | | 2.20 | |  |
| *LOH12CR1* | NM_058169.2 | Loss of heterozygosity, 12, chromosomal region 1 | | 2.57 | |  |
| *POU5F1* | NM_203289.2 | POU domain, class 5, transcription factor 1 | | -2.21 | |  |
| *PRPF19* | NM_014502.3 | PRP19/PSO4 pre-mRNA processing factor 19 homolog (S. cerevisiae) | | -2.05 | |  |
| *RBM8A* | NM_005105.2 | RNA binding motif protein 8A | | 2.46 | |  |
| *RPLP2* | BM912995 | Ribosomal protein, large, P2 | | 2.06 | |  |
| *SALL4* | NM_020436.2 | Sal-like 4 (Drosophila) | | -2.73 | |  |
| *SKP1^*^* | NM_006930.3 | S-phase kinase-associated protein 1 | | 2.70 | |  |
| *TP63* | NM_003722.4 | tumor protein p63 | | -4.35 | |  |
| *USP8* | CA312140 | Ubiquitin specific peptidase 8 | | 2.02 | |  |
| *The gene showed expression higher than 2-fold but is not included in the top network. | | | |  | |  |
