# Supplementary material for: Genome-Wide Pathway Analysis Reveals Different Signaling Pathways between Secreted Lactoferrin and Intracellular Delta-Lactoferrin
Source: PLoS One. 2013 Jan 30;8(1):e55338. doi: 10.1371/journal.pone.0055338 (PMC3559342; doi:10.1371/journal.pone.0055338)
Supplement: Table S3 — Genes in top network displaying differential expression in the ΔLF expressing cells. (DOC) [file pone.0055338.s005.doc]

| **Table S3. Genes in top network displaying differential expression in the ΔLF expressing cells** | | |  |
| --- | --- | --- | --- |
| **Symbol^*^** | **Accession** | **Description** | **Fold change** |
| *AP3D1* | NM_003938.4 | Adaptor-related protein complex 3, delta 1 subunit | 2.26 |
| *ARRB1* | BX112024 | Arrestin, beta 1 | -2.60 |
| *BARD1* | NM_000465.1 | BRCA1 associated RING domain 1 | 2.71 |
| *CLN3* | NM_000086.1 | Ceroid-lipofuscinosis, neuro--l 3, juvenile (Batten, Spielmeyer-Vogt disease) | -2.97 |
| *CSTF2* | NM_001325.1 | Cleavage stimulation factor, 3' pre-R--, subunit 2, 64kDa | 3.33 |
| *CTDP1* | NM_004715.2 | CTD (carboxy-termi--l domain, R-- polymerase II, polypeptide A) phosphatase, subunit 1 | 2.20 |
| *DDX27* | NM_017895.6 | DEAD (Asp-Glu-Ala-Asp) box polypeptide 27 | 3.10 |
| *DHX38* | NM_014003.3 | DEAH (Asp-Glu-Ala-His) box polypeptide 38 | 2.00 |
| *EFEMP2* | NM_016938.1 | EGF-containing fibulin-like extracellular matrix protein 2 | -2.74 |
| *FGD2* | NM_173558.2 | FYVE, RhoGEF and PH domain containing 2 | -2.09 |
| *FIP1L1* | NM_030917.2 | FIP1 like 1 (S. cerevisiae) | 2.06 |
| *FOXE3* | NM_012186.1 | Forkhead box E3 | 2.14 |
| *GCDH* | NM_000159.2 | Glutaryl-Coenzyme A dehydrogenase | 2.08 |
| *HEMGN* | NM_018437.3 | Hemogen | -3.44 |
| *HLA-DQA1* | NM_002122.3 | Major histocompatibility complex, class II, DQ alpha 1 | -2.74 |
| *HSPA8* | NM_006597.3 | Heat shock 70kDa protein 8 | 4.89 |
| *HSPB3* | NM_006308.1 | Heat shock 27kDa protein 3 | -2.14 |
| *KLHL20* | NM_014458.3 | Kelch-like 20 (Drosophila) | 2.42 |
| *KLK10* | NM_002776.3 | Kallikrein 10 | -2.42 |
| *MAST1* | NM_014975.1 | Microtubule associated serine/threonine kinase 1 | -2.01 |
| *MBD3* | NM_003926.5 | Methyl-CpG binding domain protein 3 | 2.00 |
| *NOS3* | NM_000603.3 | Nitric oxide synthase 3 (endothelial cell) | -2.48 |
| *NRBP1* | NM_013392.2 | Nuclear receptor binding protein 1 | 4.16 |
| *PHB2* | NM_007273.3 | Prohibitin 2 | 2.87 |
| *RIF1* | NM_018151.2 | RAP1 interacting factor homolog (yeast) | 3.77 |
| *RNPS1* | XM_041221.8 | RNA-binding protein with serine-rich domain 1 | 2.00 |
| *SALL4* | NM_020436.2 | Sal-like 4 (Drosophila) | -2.10 |
| *SKP1^*^* | NM_006930.3 | S-phase kinase-associated protein 1 | 2.60 |
| *SMN1/SMN2* | NM_000344.2 | Survival of motor neuron 1, telomeric | 2.57 |
| *SULT2B1* | NM_004605.2 | Sulfotransferase family, cytosolic, 2B, member 1 | -2.20 |
| *TCPB^*^* | NM_001140133.1 | T-complex protein 1 subunit beta | 2.80 |
| *ZNF426* | NM_024106.1 | Zinc finger protein 426 | -3.17 |
| *These genes showed expression higher than 2-fold but is not included in the top network. | | |  |
